# An Unusual Presentation of Community-Acquired Methicillin-Resistant Staphylococcus aureus Infection in a Child Treated With Linezolid

**DOI:** 10.7759/cureus.18830

**Published:** 2021-10-16

**Authors:** Amer Alshengeti, Rafid Alamri, Reem Tharwat, Hatem Alahmadi, Abdulsalam Alawfi, Maher Arkoubi, Yousef Alrashidi

**Affiliations:** 1 Department of Pediatrics, Taibah University, Al-Madinah, SAU; 2 Department of Pediatrics, Madinah Maternity and Children Hospital, Al-Madinah, SAU; 3 Department of Radiology, Madinah Maternity and Children Hospital, Al-Madinah, SAU; 4 Department of Orthopedics, Taibah University, Al-Madinah, SAU

**Keywords:** pyomyositis, linezolid, vancomycin, staphylococcus aureus, methicillin resistant

## Abstract

Methicillin-resistant *Staphylococcus aureus* (MRSA) infection is a major public health concern. MRSA isolates are classified into community-acquired MRSA (CA-MRSA) and healthcare-associated MRSA based on their epidemiology, antibiotic susceptibility patterns, and molecular characteristics. CA-MRSA typically causes skin and soft tissue infections. However, the incidence of invasive infections has increased in recent years. This paper describes the case of a 12-year-old girl with an unusual presentation of CA-MRSA. The patient presented with right thigh pyomyositis complicated by deep vein thrombosis, septic pulmonary embolism, and necrotizing pneumonia. The MRSA isolate was susceptible to vancomycin but resistant to the other anti-MRSA antibiotics. The patient was successfully treated with linezolid after clinical deterioration with vancomycin. A literature review comparing vancomycin and linezolid in invasive MRSA infections among children indicated that linezolid has better lung and tissue penetration than vancomycin, and an early switch is warranted in the case of deterioration after vancomycin administration and the lack of other alternatives.

## Introduction

Methicillin-resistant *Staphylococcus aureus* (MRSA) was recognized as a cause of nosocomial diseases in the 1960s. It was confined to healthcare settings. It was referred to as health-care-associated MRSA (HA-MRSA) [1]. In the late 1980s, MRSA cases were observed outside hospital settings and in individuals without pre-existing risk factors for HA-MRSA, whereupon it was known as community-acquired MRSA (CA-MRSA) [1]. Both CA-MRSA and HA-MRSA strains are resistant to beta-lactam antibiotics. However, CA-MRSA is often susceptible to vancomycin with variable susceptibility to other antibiotics (i.e., clindamycin, trimethoprim/sulfamethoxazole, and tetracyclines) based on epidemiology [2]. HA-MRSA strains tend to be multidrug-resistant (i.e., resistant to three or more anti-MRSA classes), and for which vancomycin is the drug of choice [1]. HA-MRSA and CA-MRSA classifications based on epidemiology alone are not accurate [2]. Isolates with HA-MRSA and CA-MRSA characteristics have been reported in community and healthcare settings, respectively [2]. Vancomycin is the preferred drug for treating invasive MRSA infections. However, herein, we report the case of an adolescent with a complicated, multidrug-resistant CA-MRSA infection who was treated with linezolid after clinical deterioration with vancomycin.

## Case presentation

**Figure 1:**
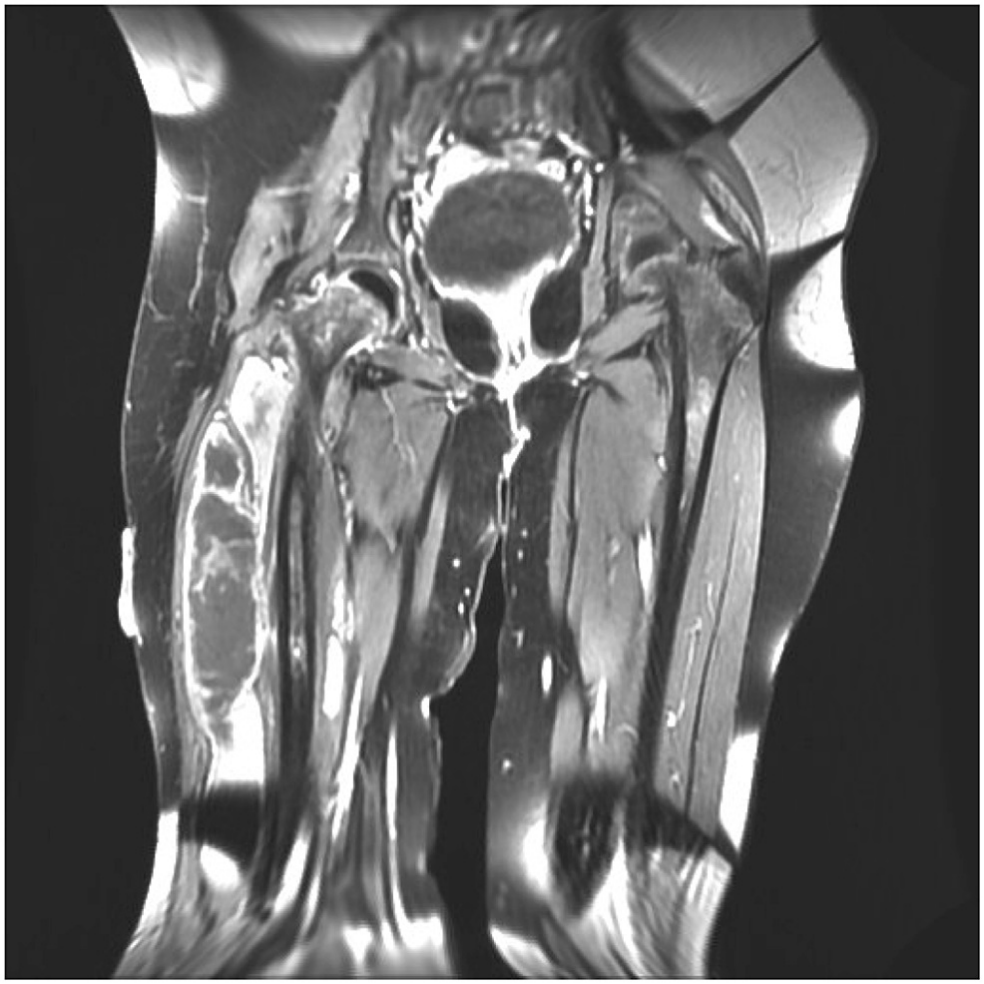
Enhanced magnetic resonance image of the right thigh and hip (sagittal view). The image shows severe subcutaneous and muscle edema with diffuse enhancement of the anterolateral muscle compartments and secondary fasciitis with no signs of septic hip arthritis or osteomyelitis.

A previously healthy 12-year-old girl was referred to our hospital with a right common femoral vein deep venous thrombosis (DVT) complicated with pulmonary embolism. Three days after a mild blunt trauma to the right thigh, she presented to the referring hospital with fever, right thigh pain, difficulty breathing, and chest pain. Doppler ultrasound of the right thigh showed DVT in the right common femoral vein. Magnetic resonance imaging of the right thigh and hip showed pyomyositis of the anterolateral muscle compartment without signs of hip joint septic arthritis or femur osteomyelitis (Figure 1). A spiral chest computed tomography scan with angiography demonstrated multiple intramural filling defects in the left lower lobe segmental branch of the pulmonary artery with multiple emboli in both lungs and a mass in the right atrium (a suspected thrombus). Therefore, the patient was administered low-molecular-weight heparin (LMWH) and transferred to our institution.

The medical histories of the patient and her family were unremarkable. The patient was fully immunized, and her developmental and growth histories were likewise unremarkable.

A physical examination in the pediatric intensive care unit indicated that the patient was overweight (weight: 53 kg, height: 139 cm, body mass index: 27.4). The patient was conscious and alert, with a blood pressure of 122/82 mmHg, mean arterial pressure of 91 mmHg, pulse of 137 beats/min, respiratory rate of 25 breaths/min, and oxygen saturation of 93% on two-liter oxygen via a nasal cannula. Chest examination revealed decreased air entry on the right side with bilateral crepitation. Cardiovascular and abdominal assessments revealed no abnormalities. Lower limb evaluation showed diffuse right thigh swelling associated with mild tenderness and a decreased range of motion at the hip joint with good perfusion. Examination of the left leg revealed normal results.

An initial laboratory work up showed a white blood cell (WBC) count of 24.6 × 10^9^/L (normal range: 4-11 × 10^9^/L) with 90% neutrophils, hemoglobin level of 8.4 g/dL (normal range: 11-15 g/dL), mean corpuscular volume of 78 fL (normal range: 80-100 fL), C-reactive protein level of 207 mg/L (normal range: 0-10 mg/L), erythrocyte sedimentation rate of 150 mm/h (normal range: 3-13 mm/h), and D-dimer concentration of 1.47 μg/mL (normal value: <0.4 μg/mL). Chest radiography showed bilateral pleural effusion, which was more on the right side. A transthoracic echocardiogram demonstrated an echogenic, non-obstructive mass in the right atrium at the inferior vena cava and the right atrium junction, suggesting an intracardiac thrombus.

After 12 hours of incubation, the blood culture grew MRSA that was susceptible to vancomycin with a minimal inhibitory concentration (MIC) of 1 μg/mL (automated VITEK®2; BioMerieux, Marcy l’Etoile, France) and 1.5 μg/mL by E-Test (BioMerieux). However, the isolate was resistant to other anti-MRSA agents (i.e., clindamycin with an MIC of >4 μg/mL, trimethoprim-sulfamethoxazole with an MIC of >4/76 μg/mL, and erythromycin with an MIC of >8 μg/mL). The antimicrobials were adjusted according to the blood culture results. The vancomycin dose was increased to 15 mg/kg/dose every six hours with a targeted trough therapeutic level of 15-20 mg/L.

Ultrasound-guided aspiration of intramuscular fluid and a muscle biopsy revealed 60 mL of turbid hemorrhagic fluid. Pathological examination showed necrotic muscle tissue with heavy infiltration of neutrophils, consistent with septic myositis. All cultures from the aspirate were negative.

The maximum vancomycin trough level reached in this patient was 11 mg/L after changing the vancomycin dosing regimen to 30 mg/kg as a loading dose followed by 20 mg/kg every eight hours on day four of treatment. 

**Figure 2:**
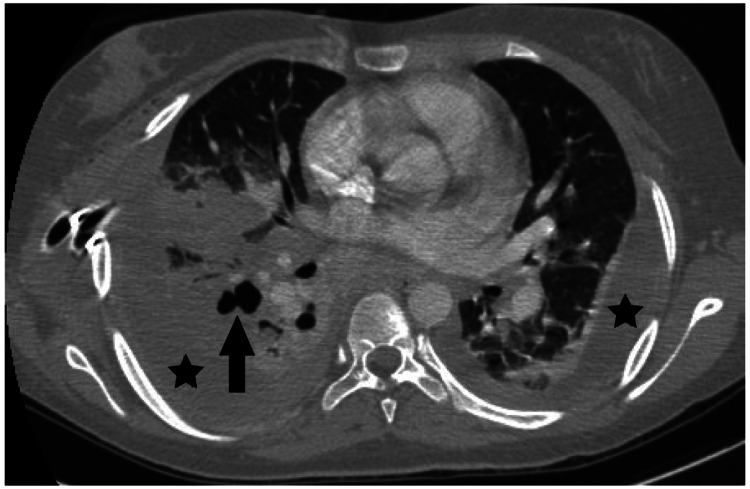
Chest computed tomography image (transverse view). The image shows bilateral pleural effusion (black stars) features of necrotizing pneumonia (multiple pneumatoceles: arrow).

Despite a negative blood culture after 48 hours, the patient continued to be highly febrile (39-40°C) and her respiratory condition worsened as the respiratory support increased to non-invasive positive pressure ventilation. The C-reactive protein level increased to 348 mg/L. Pleural effusion on the right side increased and required chest tube insertion. Pleural fluid analysis showed pleocytosis (WBC 43/μL, 90% neutrophils) with low glucose and high protein levels. However, the pleural fluid culture results were negative. Chest computed tomography showed progression to necrotizing pneumonia in the right middle and lower lobes with empyema and new septic emboli in both lungs (Figure 2). Therefore, vancomycin was replaced with intravenous linezolid (600 mg every 12 hours) after five days of vancomycin therapy.

Approximately 48 hours after starting linezolid, the fever subsided. The patient’s respiratory condition gradually improved over the next seven days. The patient was transferred to the pediatric floor with two-liter oxygen via a nasal cannula while maintaining the LMWH and linezolid.

**Figure 3:**
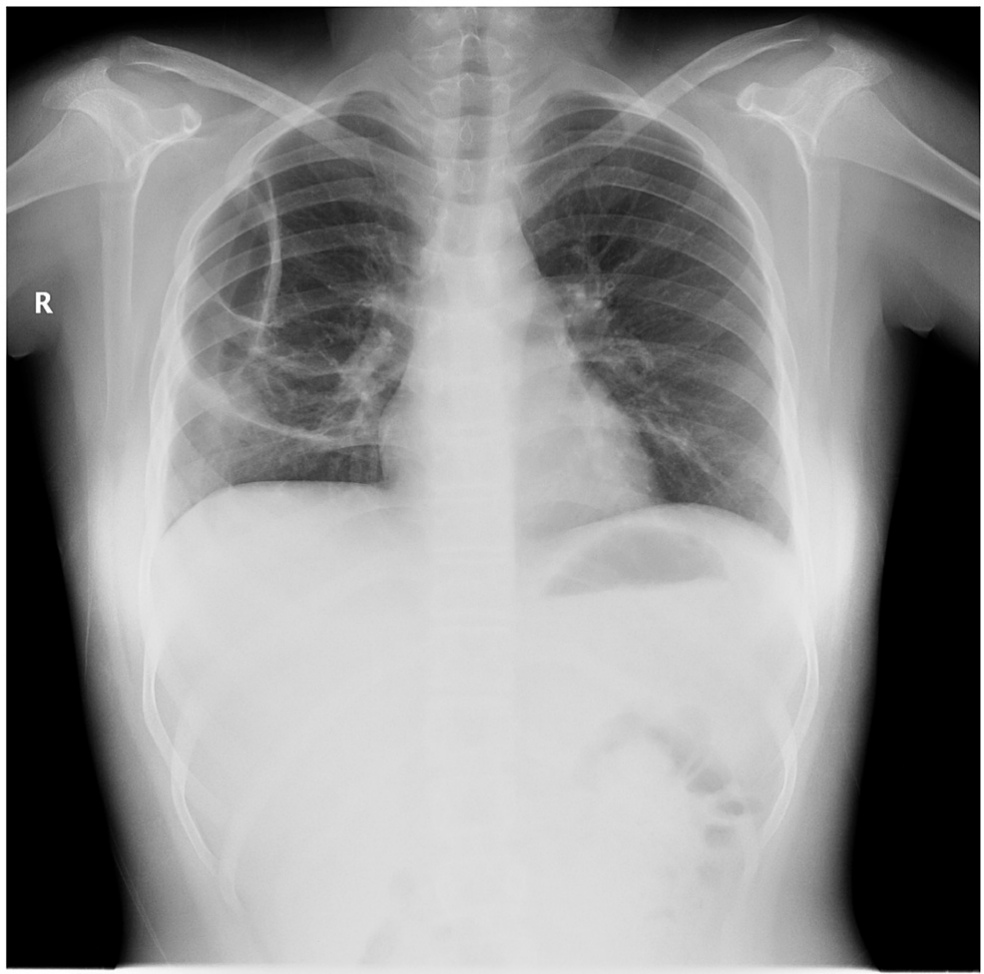
Chest radiograph (posteroanterior view). This radiograph, obtained after six weeks, shows marked improvement with remaining large pneumatoceles.

The patient’s respiratory condition continued to improve. She completed four weeks of linezolid treatment. Blood cell counts, including the WBC count, normalized and remained stable throughout the course. A follow-up chest X-ray performed one week after linezolid discontinuation showed markedly improved lung parenchyma with remaining large pneumatoceles (Figure 3). Repeated thigh magnetic resonance imaging conducted after six weeks showed resolution of the DVT and considerable pyomyositis improvement. Additionally, a repeated transesophageal echocardiogram showed resolution of the intracardiac thrombus. The patient was discharged in good condition after six weeks of hospital stay. The pneumatoceles resolved spontaneously after three months on the follow-up chest radiograph.

## Discussion

This report describes an unusual presentation of a CA-MRSA infection involving multiple sites in an immunocompetent child. The primary infection site was right thigh pyomyositis, which was complicated by DVT, intracardiac thrombosis, septic pulmonary embolism, and necrotizing pneumonia. The most common type of CA-MRSA-induced infection in children is skin and soft tissue infection [3]. However, invasive infections with severe morbidity and mortality have been reported [3]. Although there has been an increase in the incidence of pyomyositis caused by CA-MRSA over the past several years, only a few reports have linked pyomyositis with septic pulmonary embolism and necrotizing pneumonia in children [4,5]. Wong et al. [5] reported seven cases of septic pulmonary embolism associated with CA-MRSA infections. In one case, pyomyositis of the right thigh was the primary source [5].

Vancomycin is the preferred antibiotic for invasive MRSA [6]. For invasive CA-MRSA, alternatives include clindamycin and linezolid [6]. However, the isolate in this case report was resistant to clindamycin. Other anti-MRSA antibiotics (e.g., trimethoprim/sulfamethoxazole and tetracyclines) lack evidence and are thus not recommended for invasive MRSA infections [6]. Thabet et al. [7] reported a case of septic pulmonary embolism secondary to septic thrombophlebitis of the iliac vein in an 11-year-old boy who was treated with vancomycin. However, his course was not complicated with necrotizing pneumonia, unlike the patient in our case.

The vancomycin failure in the present case might be explained by the low lung concentration and the inability to reach a higher therapeutic level (>15 mg/L). A systemic review investigating the pharmacokinetics and tissue penetration of vancomycin and linezolid showed that vancomycin has low bioavailability in the lung tissue. Its concentration in epithelial lining fluid ranges from 5% to 25% of the plasma concentrations [8].

The maximum vancomycin trough level that could be reached in the patient in this case report was 11 mg/L after changing the vancomycin regimen to a 30 mg/kg loading dose followed by 20 mg/kg every eight hours. Difficulty in reaching a therapeutic level of 15 to 20 mg/L, even with a 30 mg/kg loading dose, is common in children. Demirjian et al. [9] published a randomized controlled trial (RCT) in children (2-18 years old) that compared a vancomycin loading dose (30 mg/kg) followed by 20 mg/kg/dose every eight hours (N = 19) versus conventional dosing at 20 mg/kg/dose every eight hours (N = 27). The vancomycin trough level was similar in both groups (p = 0.17), with a median level of 9 mg/L before the third dose [9].

A new method to monitor the optimum vancomycin level is the area-under-the-concentration-time-curve for 24 hours divided by the MIC (AUC24/MIC). This method may predict treatment outcomes better than the trough level when treating invasive MRSA [10]. An AUC24/MIC of >400 mg/h/L is the recommended level for severe MRSA infections [10]. There was no expert clinical pharmacist to calculate the AUC24/MIC for our patient, and data on the AUC24/MIC in children and adolescents are limited. Children have a higher glomerular filtration rate than adults, potentially making it more difficult to reach high vancomycin levels in children, especially adolescents [10]. It would be difficult to achieve an AUC24/MIC of >400 mg/h/L in serious MRSA infections, especially with an MIC ≥1 μg/mL [11].

A recent prospective study that enrolled 170 children with influenza pneumonia in intensive care units compared the outcomes of influenza with and without bacterial coinfection [12]. The authors found that among children with influenza and MRSA coinfection (N = 30), mortality was 69.2% with vancomycin monotherapy and 12.5% with double coverage using vancomycin and a second anti-MRSA antibiotic (relative risk, 5.5; 95% confidence interval: 1.4, 21.3; p = 0.003). Clindamycin was the most common second anti-MRSA drug used in this study [12].

Linezolid is a bacteriostatic oxazolidinone and works by inhibiting protein synthesis. It has excellent activity against gram-positive bacteria, including MRSA [13]. Further, it has low serum plasma protein binding (31%) with high bioavailability in most body tissues [8]. Linezolid concentrates in the lung tissue, and its level can reach double the plasma level in the epithelial lining fluid of the lung [8].

Data regarding linezolid use in invasive MRSA among children are limited to small RCTs and observational studies. One RCT comparing linezolid and vancomycin for treating invasive gram-positive infections in children showed a similar cure rate (79% vs. 74%, p = 0.36). However, there were only 30 cases of MRSA with heterogenicity at the infection site [14]. RCTs regarding linezolid in invasive MRSA infections among children, including those with necrotizing pneumonia, are lacking.

In this case, the patient tolerated linezolid, and her hemoglobin, WBC, and platelet levels remained stable throughout the treatment course. Saiman et al. [15] published a systematic review assessing the safety and tolerability of linezolid among children from four intervention studies, including two RCTs. The most common adverse events (AEs) associated with linezolid were fever, diarrhea, and vomiting, similar to groups that received the comparator drugs in the RCTs (i.e., vancomycin and cefadroxil) [15].

In adults, several AEs have been attributed to the prolonged use of linezolid (i.e., longer than four to six weeks), including hematological AEs (e.g., thrombocytopenia) and neurological AEs (e.g., peripheral neuropathy, serotonin toxicity, and ocular toxicity) [16]. However, data from osteoarticular infection studies in children showed an excellent safety profile for linezolid with a treatment duration of up to six weeks [17].

## Conclusions

This report describes a rare case of disseminated CA-MRSA in an adolescent treated with linezolid. However, we were unable to perform molecular testing to determine the isolate type (i.e., HA-MRSA or CA-MRSA), especially the presence of the Panton-Valentine leucocidin gene, which plays a major role in CA-MRSA pathogenicity. This case highlights the role of linezolid in treating invasive MRSA in children, especially cases complicated by necrotizing pneumonia. Further studies are needed to assess the effectiveness and safety of linezolid in children.
